# Patient Participation in Clinical Trials of Oncology Drugs and Biologics Preceding Approval by the US Food and Drug Administration

**DOI:** 10.1001/jamanetworkopen.2021.10456

**Published:** 2021-05-18

**Authors:** Nora Hutchinson, Benjamin Carlisle, Adelaide Doussau, Rafia Bosan, Eli Gumnit, Amanda MacPherson, Dean A. Fergusson, Jonathan Kimmelman

**Affiliations:** 1 Studies of Translation, Ethics, and Medicine, Biomedical Ethics Unit, McGill University, Montreal, Québec, Canada; 2 Centre for Practice-Changing Research, Ottawa Hospital Research Institute, Ottawa, Ontario, Canada; 3 Department of Medicine, University of Ottawa, Ottawa, Ontario, Canada

## Abstract

**Importance:**

Several studies have estimated the financial inputs for successful drug development. Such analyses do not capture the large investment that patient study participants commit to drug development.

**Objective:**

To estimate the volume of patients required to achieve a first US Food and Drug Administration (FDA) approval for a new anticancer drug or biologic therapy.

**Design, Setting, and Participants:**

This cohort study included a random sample of prelicense oncology drugs and biologics with a trial site in the United States that were launched into clinical efficacy testing between January 1, 2006, and December 31, 2010. Drugs and biologics were identified using ClinicalTrials.gov registration records. Total patient enrollment was captured over an 8-year span, and each intervention was classified based on whether it received FDA approval and was deemed as having intermediate or substantial value according to the American Society of Clinical Oncology Value Framework (ASCO-VF) score. Secondarily, the association between patient numbers and intervention characteristics was tested. Data were analyzed in February 2020.

**Main Outcomes and Measure:**

The prespecified primary outcome was the number of patients enrolled in prelicense trials per FDA approval.

**Results:**

A total of 120 drugs and biologics were included in our study, with 84 (70.0%) targeted agents, 20 (16.7%) immunotherapies, and 71 (59.2%) novel agents. A total of 13 drugs and biologics (10.8%; 95% CI, 5.3%-16.8%) in our sample gained FDA approval within 8 years, of which 1 (7.7%) was deemed of intermediate value and 3 (23.1%) were deemed of substantial value using ASCO-VF scoring. Overall, 158 810 patients were enrolled in 1335 trials testing these drugs and biologics, 47 913 (30.2%) in trials that led to FDA approval and 110 897 (69.8%) in trials that did not. An estimated 12 217 (95% CI, 7970-22 215) patient study participants contributed to prelicense trials per FDA approval. The estimated number of patients needed to produce a single FDA-approved drug or biologic of intermediate or substantial ASCO-VF clinical value was 39 703 (95% CI, 19 391-177 991).

**Conclusions and Relevance:**

The results of this cohort study make visible the substantial patient investment required for prelicense oncology drug development. Such analyses can be used to devise policies that maximize the clinical impact of research on a per-patient basis.

## Key Points

**Question:**

How many patient study participants are needed to obtain a first US Food and Drug Administration approval for a new anticancer drug or biologic therapy?

**Findings:**

In this cohort study of 120 drugs and biologic therapies, more than 12 000 patients participated in prelicense clinical trials for every new drug or biologic approved by the US Food and Drug Administration. When confined to drugs and biologic interventions with intermediate to substantial clinical impact, nearly 40 000 patients were required per approval.

**Meaning:**

These results indicate that in addition to involving large private expenses, oncology drug and biologic development entails a large subsidy of altruism, time, and welfare from patients themselves.

## Teaser

This cohort study estimates the volume of patients required to achieve a first US Food and Drug Administration (FDA) approval for a new anticancer drug or biologic therapy.

## Introduction

Recent studies estimate the median cost of bringing a new drug to market at $985.3 million for all therapeutic agents and $793.4 million for oncology drugs.^1,2^ For every 100 drugs entered into phase 1 clinical testing in oncology, fewer than 10 will ultimately receive regulatory approval.^3,4,5^ A minority of approved cancer drugs provide substantial clinical benefit.^6^

However, cost estimates and molecule success rates do not reflect the full range of inputs societies commit to pharmaceutical research. Drug development requires that patient study participants agree to submit to screening, forgo standard care, receive experimental medicines, and undergo research procedures. Drug development also requires that physicians and patients invest substantial cognitive resources in explaining and understanding study protocols. Previous studies of patient enrollment have shown that a median of 1708 patients participated in clinical trials per European Medicines Agency new molecular entity approval^7^; a median of 2316 patients were studied per US Food and Drug Administration (FDA) approval of a precision medicine oncology drug.^8^ However, these studies restricted their analyses to approved drugs and thus do not account for the large numbers of patients participating in unsuccessful drug development efforts. Estimates that include all patient study participants can make more visible the degree to which private drug development efforts build on public volunteerism. They can also help to identify research activities that more efficiently use this public endowment. The aim of this study was to estimate the number of patient study participants needed to obtain a first FDA approval for a cancer therapeutic.

## Methods

This study relies on publicly accessible data and was therefore not subject to institutional review board approval. We preregistered our study on Open Science Framework^9^; protocol deviations and explanations are outlined in eMethods 1 in the Supplement. This study follows the Strengthening the Reporting of Observational Studies in Epidemiology (STROBE) reporting guideline for cohort studies.

Our cohort consisted of drugs and biologic interventions (hereafter, drugs) with a first efficacy trial in oncology launched between January 1, 2006, and December 31, 2010. To create our cohort, we searched ClinicalTrials.gov for interventional early efficacy trials in September 2018 using the keyword *cancer* and its synonyms (eMethods 2 in the Supplement). We defined an *early efficacy trial* as a completed phase 2, phase 1/2, or seamless phase 1 trial with enrollment of at least 100 patients; we treated seamless phase 1 trails as early efficacy studies because they typically entail phase 2–like efficacy evaluation using expansion cohorts.^10^ For each unique drug identified, a zipped folder of XML files was downloaded, and relevant data fields were extracted and entered into a CSV file. FDA approval status of each drug was assessed on the FDA website.^11^ Drugs were excluded if they (1) had advanced to first efficacy trials before 2006; (2) received FDA approval in oncology before first registered efficacy trial; or (3) received FDA approval in a nononcology indication within 8 years of the first oncology efficacy trial. We focused on North American development efforts, using the presence of a US trial site as a proxy for intention to pursue FDA approval, and thus excluded drugs with no trials with a US site (eMethods 2 in the Supplement).

We chose a start date of 2006 because trial registration for efficacy studies became the norm after 2004.^12,13,14^ We anchored our cohort in efficacy studies because there is no legal requirement to register phase 1 trials.^12^ We used a 2010 close date for our cohort because it afforded at least 8 years of follow-up to determine whether interventions received FDA approval. Based on piloting, median (interquartile range [IQR]) time to new drug application (NDA) or biologics license application (BLA) among recently approved FDA drugs was 5.08 (2.96-7.58) years (eTable 1 in the Supplement). This covers a period when many new precision medicines and immunotherapies emerged.

**Figure 1.**
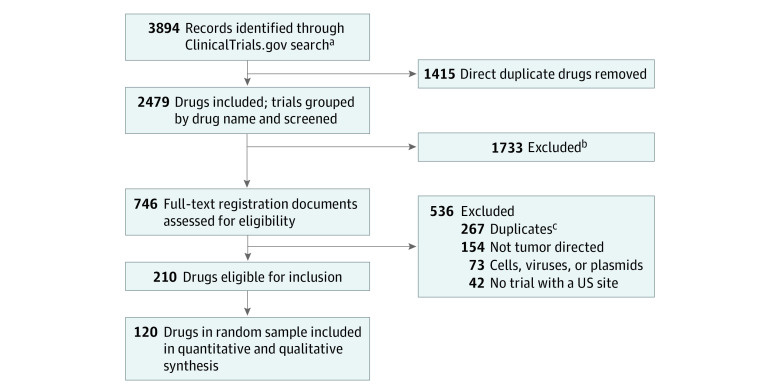
Flow Diagram for Identification of Cohort of Oncology Drugs and Biological Interventions ^a^Search of ClinicalTrials.gov for completed phase 1 trials that transitioned to a population of at least 100 from January 1, 2006, to December 31, 2010, and phase 1/2 or phase 2 trials with a launch date from January 1, 2006, to December 31, 2010. ^b^Excluded drugs were those advanced into first efficacy trial before 2006, those that received US Food and Drug Administration approval in oncology prior to first registered efficacy trial, or those that were approved for nononcology indication within 8 years of first efficacy oncology trial. ^c^Duplicate drugs were removed using drug name synonyms listed on the National Cancer Institute Thesaurus.^15^

Assessment for drug inclusion was performed by two assessors (N.H. and R.B.), with a third assessor (J.K.) resolving disagreements. The sample size was established a priori. Given the absence of historical estimates, we based our sample size on the precision of the estimated proportion of drugs that gained FDA approval among those that entered efficacy testing. Using the data of Hay et al,^3^ we calculated that 6.9% of phase 2 oncology drugs advance to FDA approval. Based on a precision of 5% for the 95% CI, using the binomial exact calculation, we sought to include at least 118 drugs in our sample. Keeping in mind study feasibility, a random sample of 120 drugs was chosen as the final cohort of investigational oncology drugs from 210 eligible candidates (Figure 1).^15^

Each drug in the cohort was subsequently assessed for drug class (eMethods 3 in the Supplement), early use of enrichment trial design (eMethods 4 in the Supplement), and novelty status (eMethods 5 in the Supplement) by 2 independent assessors (drug class and enrichment trial design, N.H. and an independent researcher; novelty status, R.B. and E.G.). A drug was classified as displaying early enrichment trial design if, in either of its first 2 oncology efficacy trials, enrollment was based on biomarker status and the drug targeted the biomarker or a molecule in its pathway. A drug was considered novel if there was no antecedent drug of the same molecular type and mechanism of action that had reached phase 3 testing. A drug was labeled as being sponsored by a large pharmaceutical company if the sponsor of either of its first 2 efficacy trials was a top 15 pharmaceutical company by revenue in the year of trial launch.

### Trial Sample and Data Extraction

For each included drug or biologic, we downloaded all prelicense oncology trials of any phase registered on ClinicalTrials.gov in March 2019, irrespective of study location. We defined prelicense as any trial initiated from the date of the first oncology efficacy study until the date of filing of an NDA or BLA for FDA-approved drugs or until 8 years of follow-up had lapsed. We excluded trials that only enrolled healthy volunteers as well as open-label extension studies (to avoid double counting patients). Because of their large anticipated enrollment and dynamic nature, patient enrollment for ongoing master protocol trials was updated, when possible, using the most recent enrollment figures available. This applied to 6 trials. Oncology patients from all eligible trials were counted toward the primary outcome, irrespective of trial status (eMethods 6 in the Supplement). Additional manual extraction of patient enrollment figures until December 31, 2019, was performed in January 2020 for drugs with 10 and 12 years of available follow-up for sensitivity analyses on our primary outcome. Extraction of early phase 1 enrollment data for all 120 drugs was also performed for further sensitivity analyses.

Cancer indications were classified by site and cell type; the 2016 World Health Organization classification was used for hematologic malignant neoplasms.^16,17^ Trial interventions were classified as monotherapy, combination therapy, or mixed modalities. For quality control, a random sample of 50 trials was evaluated for inclusion and trial classification by 2 independent assessors (N.H. and A.M.). Agreement was 98.2%; the remainder of trials was screened and classified by a single assessor (N.H.). Based on reviewer comments, we performed a post hoc assessment of orphan drug designation status for all drugs and biologic interventions in our cohort (eMethods 7 in the Supplement).

### Statistical Analysis

Our primary outcome was the number of patients required to reach a first FDA approval. We recognize that many cancer drug approvals are not based on strong evidence and that many have limited clinical value.^18^ Nevertheless, we used regulatory approval as our primary outcome because it provides a reasonable surrogate for clinical impact, and it affords a greater number of events to support statistical analysis than more meaningful impact measures, which are captured in our secondary outcomes. Our estimate for primary outcome was achieved by aggregating patient enrollment figures for all prelicense trials for the 120 drugs in our cohort and dividing by the number of FDA approvals within 8 years. A 95% CI was constructed using sampling with replacement on a per-drug basis, in which aggregate patient enrollment per drug and FDA approval status (noted as 1 or 0 for approved and nonapproved drugs, respectively) of the 120 drugs in our cohort were resampled 1000 times. This allowed us to create a Gaussian distribution; the 2.5th and 97.5th percentiles of the distribution served as upper and lower bounds of our confidence interval.

To directly measure clinical impact, we estimated the number of patients required to develop a drug of intermediate or substantial clinical value, based on the American Society of Clinical Oncology Value Framework score (ASCO-VF).^19^ ASCO-VF provides a template for assessment of treatment benefit, toxic effects, and symptom control, as demonstrated in comparative oncology clinical trials, resulting in a score of net health benefit. FDA-approved drugs that had been evaluated in completed comparative randomized clinical trials were advanced to ASCO-VF assessment. Scores were extracted from the study by Saluja et al^20^ and categorized as low (ASCO-VF score ≤40), intermediate (ASCO-VF score >40 to <45), and substantial (ASCO-VF score ≥45) benefit.^21^

We also compared the number of patients enrolled in prelicense oncology trials to reach a first FDA approval for pairs consisting of novel and not novel drugs, early enriched and not early enriched drugs, immunotherapy and not immunotherapy, drugs sponsored by a large pharmaceutical company and those not sponsored by a large pharmaceutical company, early launch (2006-2008) and late launch (2009-2010) drugs, and orphan and nonorphan drugs. This required calculating the number of patients per FDA approval and bootstrap 95% CIs for each member of a pair and using permutation testing to calculate *P* values. We provided a binomial 95% CI for the proportion of drugs reaching FDA approval. We provided median and interquartile range (IQR) estimates for patient enrollment per drug and number of trials per drug for all drugs in our sample, for drugs achieving FDA approval, and for drugs that were not granted regulatory approval within 8 years.

All analyses were performed using R version 3.6.3 (R Project for Statistical Computing). We defined *P* < .05 as statistically significant, and all tests were 2-tailed. Data were analyzed in February 2020.

## Results

**Figure 2.**
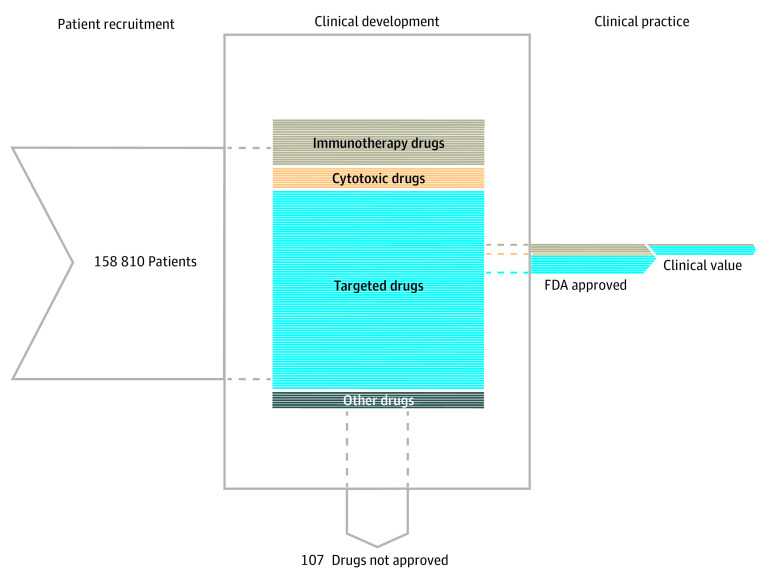
Patient Contribution and Clinical Success of Oncology Drug Development The left panel represents the patient contribution to oncology drug development, in which 158 810 patients enrolled in oncology clinical trials. The middle panel represents the clinical development of 120 oncology drugs in our sample. Each horizontal line represents a single drug, organized by drug class. The right panel represents clinical practice, in which 13 oncology drugs gained US Food and Drug Administration (FDA) approval, with 4 approved drugs deemed of intermediate or substantial clinical value by the American Society of Clinical Oncology Value Framework.

More than two-thirds of the 120 drugs in our cohort were targeted agents (84 drugs [70.0%]); 20 drugs (16.7%) were immunotherapies, and 71 (59.2%) were novel agents (Table 1). A total of 13 drugs in our cohort (10.8% [95% CI, 5.3%-16.8%]) achieved a first FDA approval within 8 years (Figure 2). Of the 13 FDA approved drugs in our cohort, 1 (7.7%) was deemed of intermediate value and 3 (23.1%) were deemed of substantial value using ASCO-VF scoring (Table 2).^19^

**Table 1.** Characteristics of Investigational Drug Cohort

Characteristic	Total drugs, No. (% of total) (n = 120)	FDA-approved drugs, No. (% by drug class) (n = 13)
Drug status at 8 y		
FDA approved	13 (10.8)	NA
Not FDA approved	107 (89.2)	NA
Drug class^a^		
Immunotherapy drugs	20 (16.7)	4 (20.0)
Targeted drugs	84 (70.0)	8 (9.5)
Cytotoxic drugs	9 (7.5)	1 (11.1)
Other	7 (5.8)	0 (0.0)
Enrichment^b^		
Early enriched	29 (24.2)	6 (20.7)
Not early enriched	91 (75.8)	7 (7.7)
Sponsor^c^		
Large pharmaceutical company	31 (25.8)	6 (19.4)
Other	89 (74.2)	7 (7.9)
Novelty^d^		
Novel	71 (59.2)	5 (7.0)
Not novel or NA	49 (40.8)	8 (16.3)
Orphan drug designation^e^		
Orphan drug	38 (31.7)	11 (28.9)
Nonorphan drug	82 (68.3)	2 (2.4)

Abbreviations: FDA, Food and Drug Administration; NA, not applicable.

^a^ See eMethods 3 in the Supplement.

^b^ See eMethods 4 in the Supplement.

^c^ A drug was considered launched by a large pharmaceutical company if 1 of its first 2 efficacy trials was sponsored by a top 15 company by revenue in the year of trial launch.

^d^ See eMethods 5 in the Supplement.

^e^ See eMethods 7 in the Supplement.

**Table 2.** Characteristics of US Food and Drug Administration–Approved Drugs

Drug	Drug class	Patient enrollment	Approval indication	ASCO-VF category (score)^a^
Afatinib	Targeted	7894	Locally advanced or metastatic NSCLC with *EGFR* variant	Low value (33.6)
Blinatumomab	Immunotherapy	1582	Philadelphia chromosome–relapsed or refractory B-cell precursor ALL	Low value (29.0)
Elotuzumab	Immunotherapy	3834	Multiple myeloma, received prior therapies	Low value (24.0)
Idelalisib	Targeted	2513	Refractory indolent non-Hodgkin lymphoma and relapsed chronic lymphocytic leukemia	Substantial value (72.0)^b^
Liposomal irinotecan	Cytotoxic	944	Metastatic adenocarcinoma of pancreas	Low value (33.0)
Moxetumomab pasudotox	Immunotherapy	157	Relapsed or refractory hairy cell leukemia	No completed comparative RCT
Necitumumab	Targeted	2315	First-line treatment for locally advanced or metastatic squamous NSCLC	Low value (16.0)
Obinutuzumab	Immunotherapy	6058	Previously untreated chronic lymphocytic leukemia	Substantial value (65.6)
Pexidartinib	Targeted	908	Symptomatic tenosynovial giant cell tumor	No completed comparative RCT
Ramucirumab	Targeted	7498	Advanced gastric or gastroesophageal junction adenocarcinoma after prior chemotherapy	Low value (22.4)
Trametinib	Targeted	3776	Unresectable or metastatic melanoma with *BRAF* variation	Intermediate value (44.0)
Vemurafenib	Targeted	4655	Unresectable or metastatic melanoma with *BRAF* variation	Substantial value (59.2)
Ziv-aflibercept	Targeted	5779	Metastatic colorectal cancer	Low value (18.3)

Abbreviations: ALL, acute lymphoblastic lymphoma; ASCO-VF, American Society of Clinical Oncology Value Framework; NSCLC, non–small cell lung cancer; RCT, randomized clinical trial.

^a^ Based on scoring of ASCO scores of the earliest trial assessed in the study by Saluja et al^20^; low (≤40), intermediate (>40 to <45), substantial (≥45) value score interpretation by Cherny et al.^21^

^b^ Approved for 2 different indications (non-Hodgkin lymphoma and chronic lymphocytic leukemia) on the same date; for the purposes of our analysis, we counted this as a single first approval and used the date of submission of the New Drug Application for non-Hodgkin lymphoma, as this was submitted prior to that of chronic lymphocytic leukemia. The ASCO-VF score is for a phase 3 trial in chronic lymphocytic leukemia, which was the only phase 3 trial performed at the time of approval.

The drugs in our cohort were tested in 1335 oncology trials (eTable 2 in the Supplement). The most common cancer indications investigated were non–small cell lung cancer (44 drugs [36.7%], studied in 173 trials), mature B-cell neoplasms (30 drugs [25.0%], studied in 103 trials), and breast cancer (37 drugs [30.8%], studied in 103 trials). The total number of patients enrolled in included trials was 158 810; 47 913 (30.2%) were enrolled in trials testing drugs that gained FDA approval, and 110 897 (69.8%) were enrolled in trials that did not. The median (IQR) patient enrollment per drug was 389 (152-1402) patients, and the median (IQR) number of trials per drug was 6 (3-14). For drugs that achieved regulatory approval, the median (IQR) number of patients was 3776 (1582-5779); for those that did not achieve approval, it was 328 (131-937) (eTable 3 in the Supplement).

Our primary outcome, the estimated number of patients enrolled in prelicense trials per FDA approval, was 12 217 (95% CI, 7970-22 215) patients. In sensitivity analyses, we reanalyzed our primary outcome for the 101 drugs for which we had 10 years of follow-up and the 58 drugs for which we had 12 years of follow-up. The number of patients enrolled in prelicense trials per new oncology drug approval was 14 774 (95% CI, 9081-29 748) patients and 11 066 (95% CI, 6775-23 150) patients for 10- and 12-year follow-up, respectively. An additional sensitivity analysis capturing all prelicense trials from phase 1 found 12 852 (95% CI, 8395-23 387) patients per first FDA approval.

**Figure 3.**
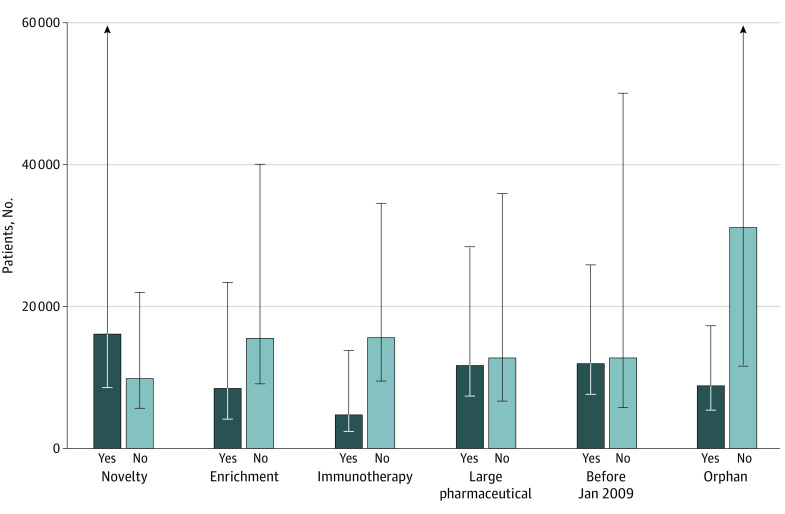
Patients Required to Develop a New Drug by Drug Property

The number of patients needed to produce a single FDA approved drug of intermediate or substantial ASCO-VF clinical value was estimated at 39 703 (95% CI, 19 391-177 991). Patient enrollment per FDA approval was 4710 (95% CI, 2395-13 748) for immunotherapies vs 15 553 (95% CI, 9456-34 430) for all others. Patient enrollment for drugs using early enrichment trial designs was 8421 (95% CI, 4115-23 310) vs 15 470 (95% CI, 9069-39 913) for non–early enrichment trial designs. Results of additional analyses of patient enrollment per FDA approval by drug property are provided in Figure 3 and eTable 4 in the Supplement.

## Discussion

To our knowledge, this study provides the first comprehensive assessment of the association between patient investment and impactful oncology drug development. It differs from prior studies that confined their assessment to successful drug development efforts.^7,8^ We estimated that 12 217 patients were required to advance a new oncology drug from initial test of efficacy to regulatory licensure. However, many drugs that gain licensure have limited clinical impact.^18^ Acknowledging wide confidence intervals owing to the small number of events, we estimated that 39 703 patients were required to develop a drug meeting ASCO-VF intermediate to substantial impact standards.

Studies of efficiency in drug development have typically focused on the financial burdens and molecule success rates of prelicense drugs. While such analyses can inform investment and policy, they miss some of the most morally salient inputs (eg, patients) and outputs (eg, clinically impactful interventions) of drug development. Measuring the association between patient contributions and impactful outputs serves 3 distinct purposes.

First, it renders the scale of patient investment in the research enterprise visible. Patient study participants, like companies, undertake risk and make substantial time commitments when they participate in prelicense trials. While ethical standards, such as clinical equipoise, ensure that patient management does not fall below the standard of care, patients nevertheless accept the risks of receiving unproven treatments, submitting to research procedures, and making additional clinical visits. This is especially true of earlier phase trials, where not participating means fewer clinic visits and procedures. While burdens may be modest at the individual level, they are substantial when aggregated across populations. In our study, the median number of patients per drug was 10 times smaller for unsuccessful prelicense oncology drug development efforts (328 patients per drug) vs drugs that achieved regulatory approval (3776 patients per drug). However, when aggregated across all drugs in our sample, 110 897 patients contributed to trials of prelicense drugs that were not granted FDA approval within 8 years. Moreover, patient contributions are largely motivated not by the prospect of helping a company commercialize a product but rather by the prospect of meaningfully advancing care of future patients. While patients may experience other direct and collateral benefits from trial participation, the magnitude of which is not addressed in our study, in our estimation, more than two-thirds of patient study participants (69.8%) contributed to research efforts that did not translate into regulatory approval within 8 years.

Assessing the association between patient volume and clinical impact can also identify research activities that offer a higher return on patient investment. While our study lacked the power for stratified analyses, exploratory secondary analyses suggest possible reduced numbers of patients needed to obtain licensure for both immunotherapy drugs (4710 vs 15 553 patients) and those using early enrichment trial designs (8421 vs 15 470 patients). The improved regulatory odds of drugs tested in biomarker enriched trials is consistent with other reports.^22,23^ If reinforced by further study, such findings should inform research priority setting. They can also be used to steer patients toward trials that are most likely to serve their goals. Of course, it will be important to put these measures of efficiency in context. For example, biomarker-enriched trials often involve a large screening burden and require valid assays to correctly identify the responsive subgroup.^24^ There are additional concerns regarding the ability of early phase biomarker trials to establish safety and efficacy, particularly when not randomized.^25^

Finally, the association between patient contributions and impactful outputs should inform research policies. For example, the 1997 Pediatric Exclusivity Provision grants a 6-month extension of market exclusivity to companies performing pediatric trials after a written request from the FDA.^26^ Drug developers have often responded to this policy by concentrating efforts on pediatric trials for blockbuster drugs rather than on drugs most likely to be impactful for pediatric patients.^27^ While the pediatric exclusivity provision has likely improved the labeling of many drugs, metrics like the ones used in our study can help to assess whether such policies have the unintended consequence of eroding moral efficiencies in pediatric clinical development.

### Limitations

This study has limitations. Enrollment in our cohort was based on the date of the first efficacy oncology study, rather than the first phase 1 trial, given that the latter are exempt from obligatory registration. Therefore, drugs that began phase 1 but never reached phase 2 are not represented in our sample. This means our estimate of patient participants represents a lower bound. Second, our study focused on first FDA approvals, not accounting for the potential impact of later approvals. This was because of our focus on the prelicense oncology drug development landscape; additional research is required to expand this analysis to the postlicense period, including assessment of subsequent FDA approvals. An integrated prelicense and postlicense analysis is also required to explore whether reduced prelicense patient enrollment comes at the cost of prolonged uncertainty and/or increased patient recruitment in postlicense confirmatory trials. Third, we performed data collection at the trial level but analyzed it at the drug level. In so doing, our estimates did not reflect the intertrial variability in patient enrollment numbers, resulting in conservative confidence intervals. Fourth, drug development efficiencies are likely affected by trends in trial design^28^ and policy changes, such as those set out in the 21st Century Cures Act.^29^ Provisions in the latter for lowering data requirements to support drug regulatory approval may result in greater efficiency, although at the expense of high-quality evidence of safety and efficacy.^30^ Follow-up studies will be required to determine whether these dynamics alter the association between patient inputs and impactful gains in cancer care. Fifth, our findings are specific to cancer drug development. Patient enrollment per trial is often relatively small,^31^ with many regulatory approvals based on single-group studies.^32^ Our approach will need to be replicated in other disease areas to estimate patient enrollment per approved drug more generally.

## Conclusions

In this study, an estimated 12 217 patients were required per FDA approval of new oncology drugs and 39 703 per FDA-approved drug with intermediate or substantial ASCO-VF clinical value. These findings indicate that oncology drug development requires substantial contributions from patient study participants, especially in the context of discovering treatments that substantially improve patient outcomes. Our findings highlight that private firms are not the only parties that bear risk, uncertainty, and opportunity cost in developing new drugs. Policy makers and research communities should strive for regulations and practices that maximize clinical impact per patient subsidy.

## Notes

Supplement. **eMethods 1.** Protocol Modifications **eMethods 2. **Cohort Identification **eTable 1.** Median Time From First Oncology Efficacy Study to BLA/NDA Submission in FDA-Approved Drugs, 2014-2018 **eMethods 3.** Assessment of Drug Class **eMethods 4.** Early Enrichment Assessment **eMethods 5.** Novelty Assessment **eMethods 6.** Example of Patient Enrollment Calculation for Blinatumomab **eMethods 7.** Orphan Drug Classification **eTable 2. **Characteristics of Included Oncology Trials **eTable 3.** Median Number of Patients and Trials per Drug **eTable 4. **Number of Patients Required to Develop a New Drug by Drug Property eReferences.
