# Supplementary material for: Patient Participation in Clinical Trials of Oncology Drugs and Biologics Preceding Approval by the US Food and Drug Administration
Source: JAMA Netw Open. 2021 May 18;4(5):e2110456. doi: 10.1001/jamanetworkopen.2021.10456 (PMC8132139; doi:10.1001/jamanetworkopen.2021.10456)
Supplement: Supplement. — eMethods 1. Protocol Modifications eMethods 2. Cohort Identification eTable 1. Median Time From First Oncology Efficacy Study to BLA/NDA Submission in FDA-Approved Drugs, 2014-2018 eMethods 3. Assessment of Drug Class eMethods 4. Early Enrichment Assessment eMethods 5. Novelty Assessment eMethods 6. Example of Patient Enrollment Calculation for Blinatumomab eMethods 7. Orphan Drug Classification eTable 2. Characteristics of Included Oncology Trials eTable 3. Median Number of Patients and Trials per Drug eTable 4. Number of Patients Required to Develop a New Drug by Drug Property eReferences. [file jamanetwopen-e2110456-s001.pdf]

Supplemental Online Content

Hutchinson N, Carlisle B, Doussau A, et al. Patient participation in clinical trials of oncology drugs and biologics preceding approval by the US Food and Drug Administration. *JAMA Netw Open.* 2021;4(5):e2110456. doi:10.1001/jamanetworkopen.2021.10456

eMethods 1. Protocol Modifications
eMethods 2. Cohort Identification
eTable 1. Median Time From First Oncology Efficacy Study to BLA/NDA Submission in FDA-Approved Drugs, 2014-2018
eMethods 3. Assessment of Drug Class
eMethods 4. Early Enrichment Assessment
eMethods 5. Novelty Assessment
eMethods 6. Example of Patient Enrollment Calculation for Blinatumomab
eMethods 7. Orphan Drug Classification
eTable 2. Characteristics of Included Oncology Trials
eTable 3. Median Number of Patients and Trials per Drug
eTable 4. Number of Patients Required to Develop a New Drug by Drug Property
eReferences.

This supplemental material has been provided by the authors to give readers additional information about their work.

© 2021 Hutchinson N et al. *JAMA Network Open.*

eMethods 1. Protocol Modifications

Modifications to secondary outcomes:
-We categorized drugs into four classes (immunotherapy, targeted, cytotoxic and other), rather than classifying drugs as small or large molecules, as the former was considered more descriptive (Modified June 2019).
-Early enrichment was added as an extraction item and for inferential testing (Modified June 2019).
-We had multiple drug approvals in our cohort and this allowed us to estimate and compare the number of patients needed to develop a new drug in novel/not novel, early enriched/not early enriched, immunotherapy/not immunotherapy, large pharmaceutical company/not large pharmaceutical company and early launch (2006-2008)/late launch (2009-2010) categories, as secondary outcomes. This was felt to provide an accurate assessment of drug development efficiency, as it relates to the patient burden of clinical testing. It therefore replaced our original intention to compare mean patient enrollment by drug category (Modified December 2019).
-Given large variation in patient enrollment figures, we reported median patient enrollment per drug and interquartile range, rather than mean patient enrollment per drug. We also reported median number of trials per drug and interquartile range.
-We performed sensitivity analyses on our primary outcome for drugs that had 10 and 12 years of follow-up (Modified October 2019; performed in January 2020 to allow for data up to 2019-12-31 to be included). We added a further sensitivity analysis to estimate total pre-license enrollment from Phase 1 divided by the number of FDA approvals (Modified January 2020).
-Based on reviewer comments, we performed a post-hoc assessment of orphan drug status, and compared the number of patients per FDA approval in orphan versus non-orphan designated drugs (Modified March 2021).

© 2021 Hutchinson N et al. *JAMA Network Open.*

eMethods 2. Cohort Identification

Using the database clinicaltrials.gov we identified early efficacy oncology drug and biologic intervention trials initiated from 2006/01/01 – 2010/12/31 inclusive using the following search criteria:

i) Condition or Disease Search terms: "Cancer OR cancers OR carcinoma OR carcinomas OR malignant OR malignancy OR malignancies OR tumor OR tumors OR tumour OR tumours OR neoplasm OR neoplasms OR metastatic OR lymphoma OR leukemia OR leukemias;"
ii) Study Type: "Interventional studies (Clinical Trials);"
iii) Status of Recruitment: "Completed;"
iv) Intervention/Treatment: "Drug or Biologic;"
v) Phase: Phase I with patient enrolment >= 100, Phase 1/2 and Phase 2
vi) Study Start Date: 2006/01/01 – 2010/12/31.

The following data elements were extracted for each identified trial: Title, Phase, Start Date, Intervention, NCTID and Locations. Trials were grouped by intervention and organized by start date.

Drug and biologic interventions were included in our cohort based on the following criteria:

We excluded the following drugs and biologic interventions from our cohort:
1. Advanced into first efficacy oncology trials before 2006
2. Received FDA approval in oncology prior to first registered efficacy trial
3. Received FDA approval in a non-oncology indication within 8 years of the first identified oncology efficacy trial (to exclude drugs repurposed for an oncology indication)
4. Aimed at primary prevention of cancer or symptoms secondary to cancer
5. Drugs or biologics that had no trials with a site in the United States
6. Devices
7. Cells, Viruses, Plasmids (generally lacking trade names in registration documents, making tracking impossible)

eTable 1. Median Time From First Oncology Efficacy Study to BLA/NDA submission in FDA-Approved Drugs, 2014-2018

Drug Name	Date 1st Efficacy Study	Date BLA/NDA Submission	Years: Trial to BLA/NDA
Tagraxofusp-erzs	1-May-13	21-Jun-18	5.08
Calaspargase Pegol-mknl	1-Nov-12	22-Dec-17	5.08
Gilteritinib	9-Oct-13	29-Mar-18	4.42
Glasdegib Maleate	27-Jun-12	27-Apr-18	5.83
Talazoparib Tosylate	16-May-14	6-Apr-18	3.83
Dacomitinib	2-Nov-07	31-Jan-18	10.17
Moxetumomab Pasudotox	15-Feb-10	29-Jan-18	7.92
Mogamulizumab	1-May-09	4-Oct-17	8.42
Encorafenib	1-Nov-12	30-Jun-17	4.58
Binimetinib	1-Apr-13	30-Jun-17	4.17
Apalutamide	4-Mar-13	9-Oct-17	4.58
Lutetium Lu 177 Dotatate	1-Oct-11	28-Apr-16	4.50
Acalabrutinib	1-Apr-15	13-Jun-17	2.17
Abemaciclib	10-Jun-14	5-May-17	2.83
Copanlisib Hydrochloride	8-May-15	16-Mar-17	1.83
Inotuzumab Ozogamicin	4-May-06	20-Dec-16	10.58
Neratinib Maleate	1-Dec-05	19-Jul-16	10.58
Durvalumab	27-Nov-13	13-Dec-16	3.00
Midostaurin	30-Jan-02	29-Aug-16	14.50
Niraparib Tosylate Monohydrate	1-Dec-09	31-Oct-17	7.83
Avelumab	6-Jan-16	23-Sep-16	0.67
Ribociclib	18-Jul-12	29-Aug-16	4.08
Rucaparib Camsylate	1-Dec-07	23-Jun-16	8.50
Olaratumab	1-Jun-09	24-Feb-16	6.67
Atezolizumab	27-Oct-09	12-Jan-16	6.17
Venetoclax	1-Nov-13	29-Oct-15	1.92
Alectinib	20-Jun-13	6-Jul-15	2.00
Elotuzumab	1-Aug-08	27-Jun-15	6.83
Necitumumab	1-Aug-07	1-Dec-14	7.33
Ixazomib Citrate	24-Aug-10	10-Jul-15	4.83
Daratumumab	26-Mar-08	9-Jul-15	7.25
Osimertinib	25-Jul-14	5-Jun-15	0.83
Cobimetinib	15-Aug-12	11-Dec-14	2.25
Trabectedin	1-Jun-02	24-Nov-14	12.42

© 2021 Hutchinson N et al. *JAMA Network Open.*

Dinutuximab	1-Aug-12	11-Apr-14	1.67
Panobinostat	1-Mar-03	22-Mar-14	11.00
Lenvatinib Mesylate	24-Jul-09	14-Aug-14	5.00
Palbociclib	1-Jan-08	13-Aug-14	6.58
Nivolumab	1-Jun-17	30-Apr-14	3.13
Olaparib	11-Jun-07	3-Feb-14	6.58
Blinatumomab	1-Jan-08	19-Sep-14	6.67
Pembrolizumab	17-Apr-12	27-Feb-14	1.83
Idelalisib	29-Sep-10	11-Sep-13	2.92
Belinostat	1-Jan-05	8-Dec-13	8.92
Ceritinib	17-Apr-12	24-Dec-13	1.67
Siltuximab	1-Aug-03	29-Aug-13	10.00
Ramucirumab	1-Nov-07	23-Aug-13	5.75
MEDIAN			**5.08**

Drugs with no completed Large Phase 1 (enrollment ≥ 100), Phase 1/2 or Phase 2 trial in oncology registered on clinicaltrials.gov prior to BLA/NDA submission were not included in this table

© 2021 Hutchinson N et al. *JAMA Network Open.*

eMethods 3. Assessment of Drug Class

Using trial registration records and drug descriptions from NCI thesaurus (https://ncithesaurus.nci.nih.gov/ncitbrowser/), with additional search of Drugbank (https://www.drugbank.ca) and PubMed (https://www.ncbi.nlm.nih.gov/pubmed/) using drug name and synonyms as required, each drug in the cohort was categorized in one of four drug classes (immunotherapy, targeted, cytotoxic or other). A permissive definition of immunotherapy was used, such that evidence of any manipulation or stimulation of the immune system to recognize and/or target cancer cells, in addition to the direct targeting of immune cells, was classified as an immunotherapy drug. Examples of immunotherapy drugs include CTLA-4 inhibitors and PD-L1 inhibitors. Targeted drugs inhibit/activate specific molecular targets, such as tyrosine kinase receptors or enzyme poly ADP ribose polymerase (PARP). Cytotoxic drugs affect all dividing cells, leading to cell death. Examples include topoisomerase II inhibitors, antimetabolites and alkylating agents. Our model was hierarchical, such that drugs with characteristics of more than one class were characterized based on their more innovative mechanism. Immunotherapy was considered the most innovative, followed by targeted therapy and cytotoxic therapy. Finally, drugs not fitting into any one of these three categories were labeled as other. For example, hormone therapy was classified as other in our study.

Duplicate assessment of drug class was carried out by two evaluators (NH & SZ) with any disagreements adjudicated by a third (JK). The first thirty drugs assessed were considered teaching cases, to ensure adequate agreement between the two primary assessors. The following ninety drugs were assessed independently, without discussion. Agreement between the evaluators using an unweighted Cohen's kappa was 0.808.

© 2021 Hutchinson N et al. *JAMA Network Open.*

eMethods 4. Early Enrichment Assessment

Our definition of enrichment was based on the definition of personalized therapy by Schwaerderle 2015 [1], in which we considered a patient population to be enriched if either i) a biomarker was used in patient selection; or, ii) no biomarker was used, but a specific patient population was selected, and at least 50% of patients with the particular disease entity are known to possess a specific biomarker. We considered a drug development program in our cohort to employ an early enrichment trial design if, in one of its first two oncology efficacy trials, the trial registration record indicated selection of an enriched patient population, and the mechanism of action of the drug targeted the specific signalling pathway (directly or one stepped removed) for which the patient population was enriched. In this way, our definition of enrichment mirrored the FDA's description of "predictive enrichment" in which a protein or genetic marker related to the drug's mechanism of action is used to identify the treatment population.[2]

Duplicate enrichment assessment was carried out by two evaluators (NH & SZ) with any disagreements adjudicated by a third (JK). The first 30 drugs assessed were considered teaching cases, to ensure adequate agreement between the two primary assessors. The following 90 drugs were assessed independently, without discussion between assessors. Agreement between the evaluators using an unweighted Cohen's kappa was 0.681.The following steps were followed in our enrichment assessment:

1. Identify the first two oncology efficacy trials for each drug using the Clinical Trials Viewer (https://trials.bgcarlisle.com/) with a full synonym list.

2. For each of the two identified trials, read the title, introduction and inclusion/exclusion criteria of their registration records on clinicaltrials.gov to determine if the patient population was enriched. Only a portion of the patient population needs to be enriched to fulfill this criterion.

3. If there is an enriched patient population, use NCI thesaurus (https://ncit.nci.nih.gov/ncitbrowser/) to determine if the drug is enriched by determining if the mechanism of action matches the selected patient population.

© 2021 Hutchinson N et al. *JAMA Network Open.*

eMethods 5. Novelty Assessment

Our novelty assessment was based on the premise that there is significant industry awareness when a pre-license drug reaches Phase 3 testing, prompting competitors to initiate clinical evaluation in similar molecules. These latter molecules, if they mirrored the type and mechanism of action of the original drug, were not considered novel. By mechanism of action we referred to the target (e.g. enzyme or receptor) of a drug which resulted in the anti-neoplastic effect. A drug in our cohort was only considered novel if one of the following conditions were met: i) no other drug with a mechanism of action that covered the main mechanism of action of the drug in our cohort was found; ii) there was no other drug of the same type (e.g. monoclonal antibody) that covered the main mechanism of action of the drug in our cohort; and, iii) drugs of the same type and with the same main mechanism of action reached Phase 3 testing (or FDA approval if no Phase 3 was conducted) only after the start date of the first oncology efficacy trial of the drug in our cohort. If none of the above criteria were met the drug in our cohort was considered not novel. If there was not enough information to determine novelty status, then a category of non-applicable was used.

Our criterion for novelty was similar to the first-in-class drug category described by Lanthier et al.[3] However, given our evaluation of a pre-license cohort of drugs and biological interventions, we used first intervention to launch Phase 3 testing, rather than first to gain regulatory approval, in our assessment.

In the following visual example, drugs X, Y and Z all have the same mechanism of action and are of the same type. Drug X is the drug in our cohort, and is considered not novel because Drug Z initiated Phase 3 testing prior to the launch of the first oncology efficacy trial of Drug X. Although Drug Y also reached Phase 3 testing, the date of launch of Drug Y's Phase 3 trial occurred after the launch of Drug X's first oncology efficacy trial. Therefore, it is Drug Z and not Drug Y than renders Drug X not novel.

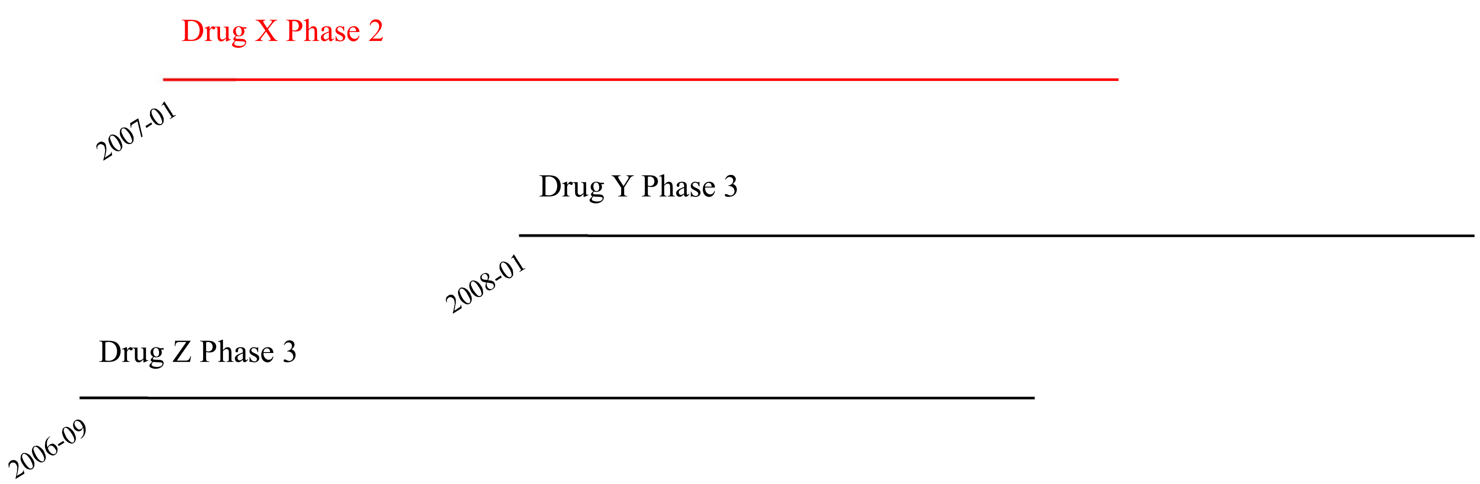

© 2021 Hutchinson N et al. *JAMA Network Open.*

Duplicate novelty assessment was carried out by two evaluators (RB & EG) with any disagreements adjudicated by a third (NH). The first twenty drugs assessed were considered teaching cases, to ensure adequate agreement between the two primary assessors. The following one hundred drugs were assessed independently, without discussion between assessors. Agreement between the evaluators using an unweighted Cohen's kappa was 0.738.

The following steps were followed to assess novelty:

1. Search NCI thesaurus (https://ncit.nci.nih.gov/ncitbrowser/) for the mechanism of action of the drug in our cohort and for its synonyms. (If no entry on NCI thesaurus, or mechanism of action unclear, also check PubChem (https://pubchem.ncbi.nlm.nih.gov/search)).

2. Perform a PubMed search of the drug in our cohort (using most common drug synonym/trade name as [Title])

3. Identify MESH terms for the drug in our cohort (selecting those which most precisely categorize the main mechanism(s) of action of the drug and type of drug (e.g. monoclonal antibody))

4. Search PubMed with the identified MESH terms, including MESH for "Clinical Trial", MESH for type of drug and MESH for mechanism of action:

-e.g. Imatinib search terms: (((fusion proteins, bcr abl[MeSH Terms]) AND protein kinase inhibitors[MeSH Terms]) AND clinical trial[MeSH Terms])

-If you do not have enough results, then remove "Clinical Trial" and repeat search

5. Beginning with the oldest PubMed articles first, identify comparator drugs with similar mechanisms of action, based on titles/abstracts from the above search.

-If there is an article which provides a review of the current drugs under investigation for a specific type of drug, then that can also be used as a useful source to identify drugs with similar mechanism of action.

6. For each new comparator drug identified, check NCI thesaurus for mechanism of action, drug type and drug synonyms. If no NCI thesaurus entry identified, then also check PubChem.

7. If similar mechanism of action and drug type, then used the Clinical Trials Viewer (https://trials.bgcarlisle.com/) with full synonym list of the comparator drug (using "OR") to determine the start date of its first Phase 3 and note down the date.

8. If similar mechanism of action and drug type, and if significant evidence of clinical trialing in the comparator drug such that FDA approval is reasonably foreseeable, using drug name and synonyms for the comparator drug, evaluate FDA approval status and date of approval by searching Drugs@FDA (https://www.accessdata.fda.gov/scripts/cder/daf/) and FDA's Biological Approvals by Year (https://www.fda.gov/vaccines-blood-biologics/development-approval-process-cber/biological-approvals-year).

© 2021 Hutchinson N et al. *JAMA Network Open.*

eMethods 6. Example of Patient Enrollment Calculation for Blinatumomab

Based on clinical trial registration records downloaded from clinicaltrials.gov, the first oncology efficacy study for Blinatumomab was identified as NCT00560794, a Phase 2 study with enrollment of 21 patients. All subsequent oncology trials were downloaded from clinicaltrials.gov from the start date of NCT00560794 (2008-01-01) until the date of filing of a Biologic License Application (BLA) for Blinatumomab, 2014-09-19 (extracted from Drugs@FDA), which occurred prior to the 8-year limit of follow-up for the primary outcome. This included nine additional trials, as demonstrated in the list below. Patient enrollment for trials in which more than one drug or biological therapy in our cohort was included in the treatment regime was evenly divided between included drugs to avoid double-counting of patients (see example of trial NCT01371630 below).

List of Trials for Blinatumomab (by order of start date of trial):

1. NCT00560794; Phase 2; 21 patients
2. NCT01209286; Phase 2; 36 patients
3. NCT01207388; Phase 2; 116 patients
4. NCT01371630; Phase 1/2; 128 patients (trial enrollment 256 patients, split evenly between Blinatumomab and Inotuzumab Ozogamicin, both therapies included in our cohort)
5. NCT01466179; Phase 2; 225 patients
6. NCT01471782; Phase 1/2; 93 patients
7. NCT01741792; Phase 2; 25 patients
8. NCT02003222; Phase 3; 488 patients
9. NCT02000427; Phase 2; 45 patients
10. NCT02013167; Phase 3; 405 patients

Pre-license patient enrollment for Blinatumomab is achieved by aggregating patient enrollment from the listed eligible trials, resulting in a total of 1582 patient-volunteers.

© 2021 Hutchinson N et al. *JAMA Network Open.*

eMethods 7. Orphan Drug Classification

As a post-hoc analysis, we assessed orphan drug designation status for all of the drugs and biologic interventions in our cohort. Using drug names and synonyms we searched https://www.accessdata.fda.gov/scripts/opdlisting/oopd/ for presence of FDA orphan designations. We classified a drug or biologic as having gained orphan status if it had ever received an orphan designation for any indication in a trial in our cohort, which was not subsequently withdrawn.

© 2021 Hutchinson N et al. *JAMA Network Open.*

eTable 2. Characteristics of Included Oncology Trials

	Number of Trials N=1335	Percent of Total (%)
Trial Status		
Completed	852	63.8
Terminated	165	12.4
Active, Not Recruiting	171	12.8
Enrolling by Invitation/Recruiting	147	11.0
Phase		
Phase 1	392	29.4
Phase 2[a]	784	58.7
Phase 3[b]	133	10.0
Phase 4[c]	8	0.6
NA	18	1.3
Randomization		
Randomized	442	33.1
Not Randomized	893	66.9
Type of Intervention		
Single Drug Therapy	589	44.1
Combination Drug Therapy[d]	697	52.2
Mixed Modalities[e]	49	3.7
Type of Enrollment		
"Actual" Enrollment	1105	82.8
"Anticipated" Enrollment	230	17.2

a) Includes Phase 1/2
b) Includes Phase 2/3
c) Phase 4 trials are trials approved by a regulatory body apart from the FDA
d) Defined as more than one anti-cancer treatment in a single trial arm
e) Consists of drug therapy in combination with surgery, radiation or other

© 2021 Hutchinson N et al. *JAMA Network Open.*

eTable 3. Median Number of Patients and Trials per Drug

	Median	Interquartile Range 25% - 75%
Median Patient Enrollment per Drug		
All Drugs	389	152 – 1402
FDA Approved Drugs	3776	1582 – 5779
Not FDA Approved	328	131 – 937
Median Number of Trials per Drug		
All Drugs	6	3 – 14
FDA Approved Drugs	16	10 – 22
Not FDA Approved	5	2 – 13

© 2021 Hutchinson N et al. *JAMA Network Open.*

eTable 4. Number of Patients Required to Develop a New Drug by Drug Property

Drug Property	Number of Patient Needed to Develop a New Drug (95% bootstrap CI)	P-Value
Novelty		
Novel	16,062 (8,536 to 61,683)	0.346
Not Novel/NA	9,813 (5,639 to 21,915)	
Enrichment		
Early Enrichment	8,421 (4,115 to 23,310)	0.353
No Early Enrichment	15,470 (9,069 to 39,913)	
Drug Class		
Immunotherapy	4,710 (2,395 to 13,748)	0.232
Not Immunotherapy	15,553 (9,456 to 34,430)	
Sponsor		
Large Pharma	11,643 (7,355 – 28,207)	0.886
Not Large Pharma	12,708 (6,648 – 35,815)	
Year of Trial Launch		
Pre-2009	11,909 (7,594 – 25,779)	0.903
2009-2010	12,709 (5,730 – 50,838)	
Orphan Drug Designation		
Orphan Drug	8,796 (5,372 – 17,222)	0.043
Non-Orphan Drug	31,027 (11,555 – ∞)	

© 2021 Hutchinson N et al. *JAMA Network Open.*

eReferences.

1. Schwaederle M, Zhao M, Lee JJ, et al. Impact of Precision Medicine in Diverse Cancers: A Meta-Analysis of Phase II Clinical Trials. *J Clin Oncol.* 2015;33(32):3817-3825.
2. Enrichment Strategies for Clinical Trials to Support Determination of Effectiveness of Human Drugs and Biological Products: Guidance for Industry. U.S. Department of Health and Human Services Food and Drug Administration. 2019. (https://www.fda.gov/media/121320/download). Accessed 12/12/2019.
3. Lanthier M, Miller KL, Nardinelli C, Woodcock J. An improved approach to measuring drug innovation finds steady rates of first-in-class pharmaceuticals, 1987-2011. *Health Aff (Millwood).* 2013;32(8):1433-1439.

© 2021 Hutchinson N et al. *JAMA Network Open.*
